# Quo Vadis PCA? A Review on Current Concepts, Economic Considerations, Patient-Related Aspects, and Future Development with respect to Patient-Controlled Analgesia

**DOI:** 10.1155/2020/9201967

**Published:** 2020-02-13

**Authors:** S. Nardi-Hiebl, L. H. J. Eberhart, M. Gehling, T. Koch, T. Schlesinger, P. Kranke

**Affiliations:** ^1^Department of Anesthesia and Intensive Care, University Hospital Marburg, Marburg, Germany; ^2^Department of Anaesthesia and Critical Care, University Hospitals of Wuerzburg, Wuerzburg, Germany

## Abstract

This review assesses four interrelating aspects of patient-controlled analgesia (PCA), a long-standing and still widely used concept for postoperative pain management. Over the years, anaesthesiologists and patients have appreciated the benefits of PCA alike. The market has seen new technologies leveraging noninvasive routes of administration and, thus, further increasing patient and staff satisfaction as well as promoting safety aspects. Pharmaceutical research focuses on the reduction or avoidance of opioids, side effects, and adverse events although influence of these aspects appears to be minor. The importance of education is still eminent, and new educational formats are tested to train healthcare professionals and patients likewise. New PCA technology can support the implementation of efficient processes to reduce workload and human errors; however, these new products come with a cost, which is not necessarily reflected through beneficial budget impact or significant improvements in patient outcome. Although first steps have been taken to better recognize the importance of postoperative pain management through the introduction of value-based reimbursement, in most western countries, PCA is not specifically compensated. PCA is still an effective and valued technique for postoperative pain management. Although there is identifiable potential for future developments in various aspects, this potential has not materialized in new products.

## 1. Introduction

Since more than 50 years, anaesthesiologists in the developed world leverage the benefits of patient-controlled analgesia (PCA) for postoperative pain management. In the mid-1960s, Philip Sechzer, an American anaesthesiologist, developed the concept whereby the patient initiates the administration of pain medication on demand depending on the perceived level of pain [1]. Over the years, PCA has been well-established as a routinely used therapy option in hospitals with different routes of administration [2, 3]. Compared to nonpatient-controlled analgesia modalities, patients report a higher satisfaction and better pain control [4]. The technique has also been associated with the potential to lower healthcare cost and shortening the length of stay in hospitals [5].

Despite the rise of new postoperative analgesic techniques like long-acting regional analgesia or continuous wound infusion together with a vivid discussion about benefits and drawbacks of PCA [6], national guidelines include specific recommendations for the use of PCA and it remains an essential component of today's therapeutic portfolio [7, 8]. However, the rate of utilization varies significantly between countries due to various reasons [9, 10]. We therefore reviewed four major aspects comprising the PCA concept and considered current and prospective developments for improvement.

### 1.1. PCA: A Multifaceted Concept

PCA consists of a technology to control the administration of a drug, which is the primary component of action. The technique requires staff and patient interaction to facilitate and operate PCA. Cost-effectiveness is the cumulating result parameter to allocate budgets and facilitates the use of the concept.

**Figure 1:**
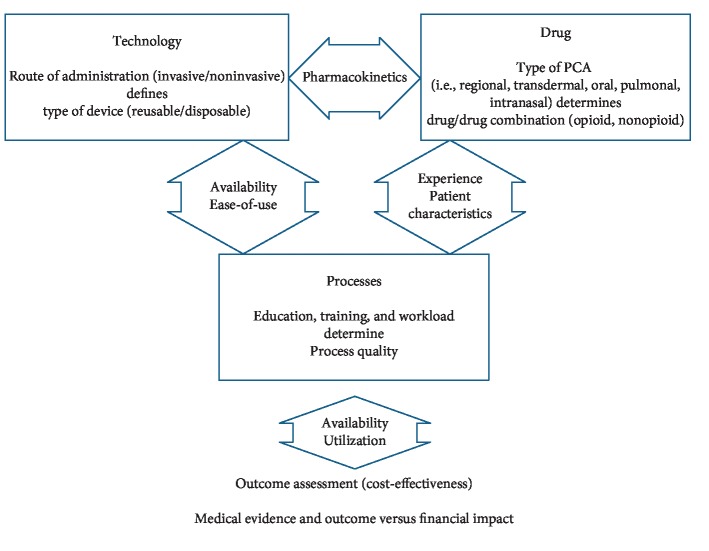
PCA conceptual framework comprising four aspects.

We therefore discuss the current state and potential developments in different areas based on a conceptual framework comprising four aspects (Figure 1):

- Technology: the technological system to control the administration of a pharmaceutical substance as well as all required material to deliver the drug into the human body
- Pharmaceuticals: the pharmaceutically active substance interacting with the human body
- Processes: the required activities to educate, to initiate, to maintain, and to conclude the therapy
- Outcome: the overall cost-effectiveness perspective of PCA

The simple reduction of PCA towards the two major components, technology and drug, does not cover the entire picture. The quality of the associated processes is a significant driver for the success or failure of the therapy [11–14]. Next to material costs and depreciation, costs incurred through actions required due to adverse events and staff costs are weighted against budgets as well as revenue potential. While the primary decision factor for use is the anticipated effectiveness after weighting potential adverse effects and safety concerns for a specific patient, we believe the economic impact finally governs the availability and utilization of PCA.

### 1.2. Technology: Safety as Its Core!

PCA technologies exist for invasive and noninvasive administration. A further distinction can be made whether the device is reusable or is disposable. While all reusable devices incorporate electronics to control functions, this is not necessarily the case with disposables. The technology and construction of the device is inevitably linked to the route of administration.

The most common device utilized for PCA is a reusable pump delivering a drug through a catheter into the human body and thus requires an invasive procedure. The pump is prepared, a drug reservoir filled or attached and used either as intravenous, subcutaneous, or regional (peripheral or central neuraxial) PCA. Current PCA practices predominantly rely on the administration of opioids with the associated harms of the drug class through misuse and overdosing. Hence, the utmost important feature of the device is the capability to control the administration in relation to time and volume to avert overdosing.

The design and construction of the pump shall principally prevent the unauthorized access and misuse of the drug. The incorporation into or the attachment of the drug reservoir to the device is intended to be tamper proof; however, this does not exclude the possibility to forcefully destroy the device and remove the reservoir. Likewise, the device does not avert the diversion of the drug through the catheter.

Electronic pumps are prone for technical and human errors. Between 2005 and 2009, the FDA recorded more than 710 deaths related to infusion pumps, specifically citing drug overdoses as one cause [15]. A recent review of the FDA Manufacturer and User Facility Device Experience database by Lawal et al. focusing on the intravenous administration route concluded that “patients on IV-PCA continue to experience serious complications as a result of preventable errors” [16]. However, changes in the design, improved user interfaces, and tightening of regulations might have led to a decrease of incidents compared to years at the beginning of the century although an increase in reporting due to legal requirements in recent years could contradict this hypothesis. In the event of a device issue, the majority of patients suffer from adverse events, whereby inadequate analgesia dominates [16, 17].

Disposable devices share certain safety issues like misuse or human handling errors, but they circumvent the burden of cleaning, maintenance, and as such the potential source of pathogen cross-contamination [18, 19]. The devices are usually elastomeric pumps or pumps based on the concept of a syringe incorporating a strong spring. These devices are almost exclusively applied for regional analgesia as they continuously administer the drug. Various pumps offer the possibility to attach a separate reservoir to provide bolus administration, thus mimicking PCA functionality. The refill mechanism of the bolus reservoir is based on an elastic balloon; however, this does not prevent the patient to administer a bolus continuously, which needs to be considered concerning the concentration when preparing the drug. As the reliability of elastomeric pumps is debated [20, 21], new developments for such disposable pumps include electronic monitoring systems which can be attached to the device [22].

While reusable and disposable pumps are exclusively for parenteral delivery, other devices offer noninvasive drug delivery without the need of a catheter. In 2008, a disposal electronic transdermal system had been introduced to the markets in the United States and Europe (IONSYS®, The Medicines Company, Parsippany/New Jersey, United States of America) [23]. The system was a preprogrammed delivery system based on iontophoresis and contained electronics as well as a drug unit. The patient activated the system on demand, and the system delivered a fixed fentanyl dose when the request was made outside of a lockout interval. After 24 hours, the system needed to be replaced. The device was withdrawn from the market in 2008 due to technical issues [24]. After reengineering, it was briefly reintroduced in 2016, but again withdrawn shortly afterwards due to commercial reasons.

In 2015, another noninvasive, reusable system has been introduced to the European market: a dispenser for sublingual tablets in combination with sufentanil-containing bioadhesive nanotablets (ZALVISO®, Grünenthal GmbH, Aachen, Germany). The system incorporates a cartridge with nanotablets containing 15 *μ*g sufentanil, and the system electronics control the dispensing of single tablets through a lockout interval [25]. A radiofrequency identification thumb tag links the device to a specific patient to prevent misuse. However, the system cannot frustrate the collection of tablets for malicious purpose.

Both noninvasive systems have shown advantages concerning postoperative mobilization. Avoiding cables and catheters positively impact the psychology of the patient and allows early and uncomplicated mobilization of the patient in the early postoperative period.

Other PCA device concepts for various routes of administration have been suggested like reusable dispensers to take medication like liquids [26] or standard pills [27] orally, administer opioids through inhalation [28, 29], or administer the drug through the nose [30, 31]. These concepts have either not gained significant market traction or have not been commercially developed yet.

### 1.3. Pharmaceuticals: Fast Onset with No Adverse Events!

Depending on the route of PCA administration, a variety of drugs is at the anaesthesiologist's disposal. For intravenous administration, common opioids like morphine, fentanyl, hydromorphone, oxycodone, or tramadol are used. Research is conducted to what extend drug combinations, for example, with ketamine [32], have positive aspects and whether it may be beneficial to add antiemetics [33, 34]. For regional techniques, a combination of opioids and long-acting local anaesthetics (e.g., bupivacaine and ropivacaine) provide high efficacy, and additional additives like clonidine or dexmedetomidine are added to prolong the effect of local anaesthetics [35]. Sufentanil is administered in combination with a dedicated dispenser for sublingual administration [25], and fentanyl had been used in conjunction with a transdermal system [23]. Fentanyl has also been studied for intranasal administration [30, 36].

Whether the overall success of a PCA therapy specifically depends on the drug used is for debate. The selection will likely be based on the individual experience of the anaesthesiologist, specifics of the hospital, and patient-related factors. Nonetheless, it is important to consider the route of administration in relation to pharmacokinetic parameters of the drug. For quick pain relief, a fast onset time of effect is desirable. Intravenous administration provides advantages compared to other routes. However, it must be considered in relation to the lockout time settings of the device to avoid dose stacking or patient dissatisfaction.

Side effects are not necessarily related to the choice of a particular opioid. The most common side effects of opioids include nausea and vomiting, pruritus, and to a lower extend sedation and respiratory depression. A recent review by Dinges et al. concludes that the choice of the opioid has only little effect on the incidence of pruritus, nausea, and vomiting [37]. In addition, the study also found the highest patient satisfaction scores with oxycodone and fentanyl and its analogues alfentanil and remifentanil. The good scores of lipophilic opioids may be viewed as an indication for the fact that onset time plays a crucial role for patient satisfaction.

The opioid side effect causing the most concern is respiratory depression. Risk factors include age of the patient and preexisting diseases. Factors associated with the occurrence of respiratory depression include the concurrent use of a back-ground infusion, advanced age, concomitant administration of sedative/hypnotic medications, and preexisting sleep apnoea syndrome [38, 39]. On the contrary, a guideline-conform intravenous PCA appears to cause no significant respiratory depression [40].

Fuelled by the so-called opioid crises in various countries, the search for nonopioid drugs to use within the PCA concept is afoot. Although any contribution of PCA, administered in a controlled hospital setting under supervision of healthcare professionals, to these crises is hard to imagine, recent research has been focused on opioid/nonopioid drug combinations [41, 42] to reduce opioid consumption or on nonopioids [43] to avoid opioids altogether.

### 1.4. Processes: Reliable and Efficient!

The associated processes contribute considerably to the success of the concept. Although the applied technology and administered drug stipulate the required actions, changes in the work environment, staffing situation, or patient characteristics influence the workflow, positively or negatively.

Managing the educational process is one of the most important aspects to deliver PCA safely and communicate appropriately to patients. Due to the nature of the involved technology, providing training for healthcare professionals to use the device and the required material is mandatory but apparently not always sufficient [44]. The importance of device-related training becomes obvious when considering the associated cost of USD 551 per device error [45]. Particular focus is required to triage patients for PCA and consecutively monitor them. Paying consideration to a patient's cognitive as well as haptic and motoric ability, age, as well as morbidity status needs to be trained. While conventional class training is predominant, new techniques like simulation training for healthcare professionals have been studied with success [46, 47].

Work-related factors also influence the quality of process outcomes. It has been suggested that aspects like emotions, professional recognition, and work load do have an influence [48]. While emotions depend on the individual's situation and hospitals as employers might provide programs to cope with such conditions, the nursing profession suffers a lack of attractiveness with impact on motivation [49]. Staff shortages apparent in most western countries reflect this situation to a large extend. PCA therapies are known to require significant staff time with a varying degree depending on the technology involved. It is therefore interesting to note that noninvasive PCA technologies with less staff time requirements lead to a higher staff satisfaction; however, this relationship has not been directly studied [50–53].

Positive effects on patient mobility can be noted when patients receive sufficient analgesia coupled with avoidance of cables, wires, and catheters.

Education of the patient is also an important element, specifically empowering the patient to leverage the major benefit of PCA: to efficiently control the pain without any fears. As paediatric patients are also able to use PCA to effectively and safely control their pain, the involvement of parents in the educational process is especially important for very young patients to keep the therapy safe [54, 55].

### 1.5. Finances: Within the Budget!

Scrutinizing cost and investment of treatment options is a common practice during times of tight budgets. Most healthcare systems in developed countries around the world feel some sort of cost pressure and introduce assessments to determine cost-effectiveness of therapies to allocate funds. Hence, questions are raised whether PCA is a cost-effective concept to treat postoperative pain management. At first glance, the simple injection of morphine intramuscularly or the administration of long-acting oral pain medication appear much more efficient than using PCA. However, depending on the initial indication for surgery, the type of PCA, national cost schedules, and the chosen comparator, it is selected whether PCA is cost-effective or not [56–59]. As these factors vary, a universal statement is not viable.

Next to cost, reimbursement plays an important role in the adoption of a practice. In most countries, specific reimbursement for PCA or postoperative pain management in general is nonexistent. In some countries, partial reimbursement is available (regional anaesthesia in the United States serves as an example), but the majority of healthcare systems do not provide specific cost coverage for postoperative pain therapy, especially in the case of a DRG lump sum payment system.

This situation does not necessarily promote the widespread use of PCA for all suitable patients as current PCA options come with significant cost defined by four elements: costs for the technology, drugs, staff, as well as adverse events and side effects. Theoretically, a higher patient satisfaction through PCA can lead to better marketing and attraction for a hospital, resulting in increased revenue potential, but many healthcare systems budget the payments towards hospitals. An offset of cost is thus unlikely. This attitude may change if new policies are implemented, emphasizing the approach of value-based reimbursement [60]. In view of this trend metrics, incorporating the effective management of postsurgical pain and postoperative nausea and vomiting become increasingly important [61].

Considering newly introduced PCA technologies, discussions might arise whether the associated price tags for apparently innovative products justify the attributed medical and practical improvements. The cost for PCA drugs as such is not perceived as prohibitive, and most hospitals have PCA-ready devices on the shelf anyway. But PCA devices are staff-intensive: the associated workload and thus associated staff cost might influence the decision for or against PCA.

### 1.6. The Way Forward

A new PCA technology would benefit from further safety aspects, deterring the accumulation of the drug as well as finding a balance between hygienic aspects, favouring disposals, and environmental issues. Also the combination of monitoring patient parameters and a feedback loop to the device might be beneficial. The route of administration shall be noninvasive, allowing the mobilization of patients quickly after the surgical procedure. Technology must be easy to use in any respect to reduce the potential for device and human error. As the drug needs to fit the route of administration, a commonly used drug with the appropriate pharmacokinetics and potency is suitable. Simple and efficient handling through preassembled components supports a high process quality as well as an intuitive operation for healthcare professionals and patients to speed up education and training. Finally, an attractive price is the basis for a wide and rapid adoption of this new solution.  Table 1 summarizes various ideas.

Next to technological and pharmaceutical advances, further trials should be conducted and focus on specific aspects. While most efficacy trials incorporate pain scores as the primary end point, a wider consideration of the transsectoral impact of utilizing PCA technology for pain management is desirable. This might strengthen the case of utilizing a more resource-intensive technique in hospitals to gain benefits later on. With the potential integration of new technologies for smart and real-time monitoring, trails investigating the individual adjustment of the PCA bolus regime in relation to bolus requests or the real-time adjustment of the lockout interval as well as dosing shall be considered.

## 2. Conclusion

Patient-controlled analgesia is an effective treatment option for postoperative pain management appreciated by healthcare professionals and patients. Current developments concentrate on noninvasive routes of administration, drug combinations, or new formulations to reduce or avoid opioid use as well as improve education and training to reduce associated adverse events through or human errors. A significant new development is not imminent.

The PCA concept has several advantages: it empowers the patient, it is effective, side effects are usually minor and adverse events rare, it can be well managed, and it is cost-efficient for defined conditions. Various routes of administration exist, predominated by intravenous and regional techniques, and variety of drugs with different characteristics are available. Current research and development is primarily concerned with drug combinations and the use of nonopioids as well as replacement therapy options like continuous wound infiltration. The importance of education is stressed, and the economic impact assessed. While all these issues are of importance, a review of the accessible and latest publication does not lead to the belief that a significant new development is imminent. This is unfortunate in the respect that room for improvement is certainly present.

Whether such a solution or any other innovative pain management solution will ever see the market also depends on the importance anaesthesiologists attribute towards guideline-conform and evidence-based treatment for postoperative pain and utilizing PCA where superior.

## Figures and Tables

**Table 1:** Summary of research and development potential.

Aspect	Current issues	Imaginable developments and activities
Technology	(i) Safety (catheter-related and tablet diversion)	(i) Noninvasive administration without the potential of collectability of drug
	(ii) Invasive	(ii) Balance between contamination potential (prodisposal) and environmental aspects
	(iii) Contamination	(iii) Additional device safety features to counter side effects and adverse events
		(iv) Real-time monitoring
Pharmaceuticals	(i) Reduction or avoidance of opioids	(i) Assessment of nonopioids with fast onset and adequate potential for pain relief
	(ii) Long-acting local anaesthetics	(ii) Drug combinations to reduce the potential for side effects and adverse events
Processes	(i) Workload related to technology and route of administration	(i) Simplification of technology by considering noninvasive routes of administration
	(ii) Education	(ii) Introduction of new educational formats
Financials	(i) Reimbursement linked to patient outcome	(i) Education of budget decision makers concerning the importance of pain management in relation to patient outcome
	(ii) Cost of new products	(ii) Understanding within the supplying industry that only a measurable value-add leads to market adoption
